# Three-Dimensional Flow of an Oldroyd-B Fluid with Variable Thermal Conductivity and Heat Generation/Absorption

**DOI:** 10.1371/journal.pone.0078240

**Published:** 2013-11-04

**Authors:** Sabir Ali Shehzad, Ahmed Alsaedi, Tasawar Hayat, M. Shahab Alhuthali

**Affiliations:** 1 Department of Mathematics, Quaid-i-Azam University, Islamabad, Pakistan; 2 Nonlinear Analysis and Applied Mathematics (NAAM) Research Group, Faculty of Science, King Abdulaziz University, Jeddah, Saudi Arabia; Bascom Palmer Eye Institute, University of Miami School of Medicine, United States of America

## Abstract

This paper looks at the series solutions of three dimensional boundary layer flow. An Oldroyd-B fluid with variable thermal conductivity is considered. The flow is induced due to stretching of a surface. Analysis has been carried out in the presence of heat generation/absorption. Homotopy analysis is implemented in developing the series solutions to the governing flow and energy equations. Graphs are presented and discussed for various parameters of interest. Comparison of present study with the existing limiting solution is shown and examined.

## Introduction

Investigation of non-Newtonian fluids in recent time has received much attention of the researchers for their industrial and engineering applications. In particular these fluids are important in material processing, chemical and nuclear industries, geophysics, bioengineering, oil reservoir engineering, polymer solutions etc. It is well known that all the non-Newtonian fluids on the basis of their behavior in shear cannot be described by a single relationship between the shear stress and shear rate. Therefore many models of non-Newtonian fluids exist. Such models are based either on natural modifications of established microscopic theories or molecular considerations. The complexity of constitutive equations in the non-Newtonian fluids is the main culprit for the lack of analytical solutions in general. Even such complexity also offer interesting challenges to the computer scientists, mathematicians and engineers for the numerical solutions. Amongst the several models of non-Newtonian fluids, the Oldroyd-B is one which can takes into account the relaxation and retardation times effects [1]–[10].

The boundary layer flow induced by a stretching surface has importance in the aerodynamic extrusion of plastic sheets, crystal growing, continuous casting, glass fiber and paper production, cooling of metallic plate in a bath, the boundary layer along a liquid film in the condensation process and many others. Such consideration in presence of heat transfer has central role in the polymer industry. In such processes, the quality of final product greatly depends upon the cooling rate and kinematics of stretching. Crane [11] firstly presented exact analytic solution for the two-dimensional boundary layer flow of viscous fluid over a linearly stretching surface. Later, this problem later has been extensively examined through various aspects of stretching velocities, suction/blowing, magnetohydrodynamics, heat/mass transfer, non-Newtonian fluids etc (see few recent articles regarding to two- and three-dimensional flows [12]–[20]). Further the concept of heat generation/absorption is useful in the cases involving heat removal from nuclear fuel debris, underground disposal of radioactive waste material, storage of food stuffs and dislocating fluids in packed bed reactors.

All the above mentioned articles deal with the fluids with constant thermal conductivity. However in reality the thermal conductivity changes with the temperature. To our knowledge, no attempt has been made for the three-dimensional boundary layer flow of an Oldroyd-B fluid with variable thermal conductivity. Even such attempt for Maxwell fluid is not available. In this work, the conservation laws of mass, momentum and energy are reduced to nonlinear ordinary differential systems. The outcoming problems are solved by homotopy analysis method (HAM) [21]–[29]. The velocity components and temperature are analyzed through their graphical representations. Local Nusselt number is examined with the help of tabular values.

### Governing problems

We consider the steady three-dimensional flow of an incompressible Oldroyd-B fluid. The flow is caused by a stretched surface at 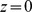. The flow occupies the domain 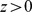. The ambient fluid temperature is taken as 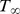 . The thermal conductivity is a linear function of temperature. Boundary layer flow is considered in the presence of heat generation or absorption. The governing equations for three-dimensional flow and heat transfer are as follows:
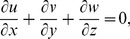
(1)

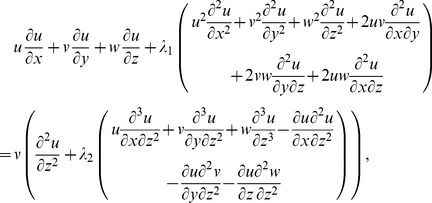
(2)

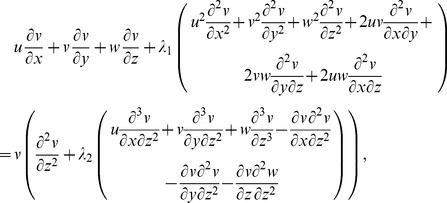
(3)

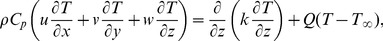
(4)where the respective velocity components in the 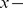, 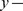 and 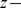 directions are denoted by 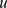, 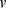 and 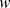, 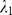 and 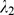 show the relaxation and retardation times respectively, 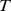 the fluid temperature, 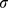 the thermal diffusivity of the fluid, 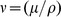 the kinematic viscosity, 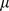 the dynamic viscosity of fluid, 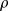 the density of fluid and Q the heat generation/absorption parameter.

The subjected boundary conditions are
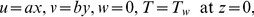
(5)

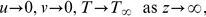
(6)in which 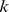 is the thermal conductivity of fluid and 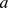 and 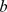 have dimensions inverse of time.

Expression of variable thermal conductivity is
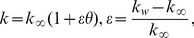
(7)where 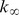 is the fluid free stream conductivity and 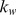 the conductivity at the wall.

The following transformations are utilized to facilitate the analysis:
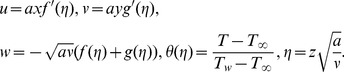
(8)Now Eq. (1) is satisfied automatically and Eqs. (2)–(7) yield
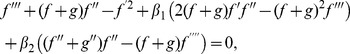
(9)

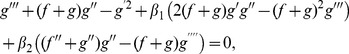
(10)

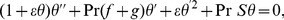
(11)

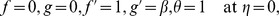


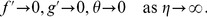
(12)In above expressions, 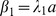 and 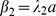 are the Deborah numbers 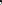 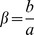 is a ratio of stretching rates parameter, 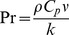 is the Prandtl number and 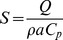 is the heat generation/absorption parameter.

The local Nusselt number with heat transfer 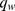 is defined as follows:
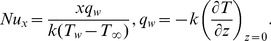
(13)Dimensionless variable reduce the above equation in the following form
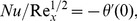
(14)where 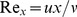 is the local Reynolds number.

### Series solutions

Initial approximations and auxiliary linear operators for homotopy analysis solutions are selected in the following forms:
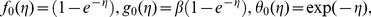
(15)

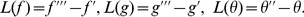
(16)The above operators have the properties
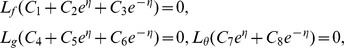
with 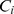 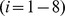 as the arbitrary constants.

The associated zeroth order deformation problems can be written as
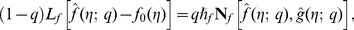
(17)

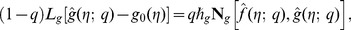
(18)

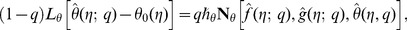
(19)

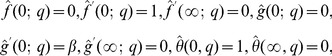
(20)

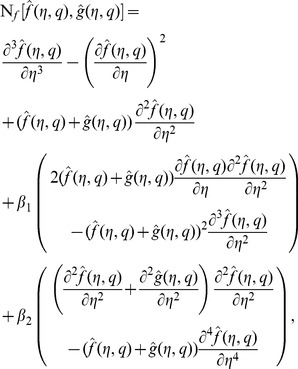
(21)

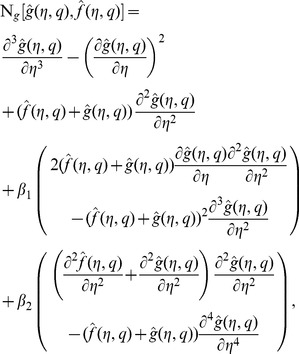
(22)

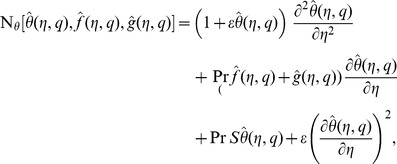
(23)in which 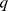 is an embedding parameter, 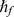, 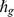 and 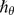 the non-zero auxiliary parameters and 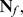 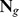 and 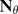 the nonlinear operators. For 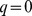 and 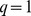 we have
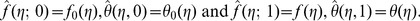
(24)When 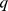 increases from 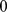 to 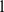 then 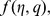 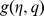 and 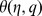 vary from 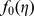, 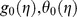 to 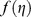, 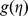 and 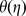 respectively. By Taylor series one obtains
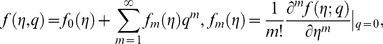
(25)

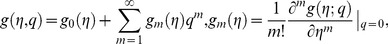
(26)

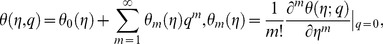
(27)where the convergence of above series strongly depends upon 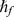, 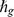 and 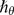. Considering that 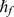, 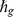 and 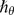 are selected properly so that Eqs. (17)–(19) converge at 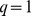 then
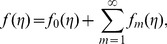
(28)

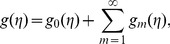
(29)

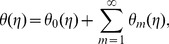
(30)and the general solutions are given by
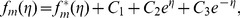
(31)

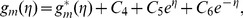
(32)

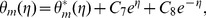
(33)in which the 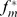, 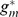 and 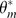 show the special solutions.

## Analysis

**Figure 1:**
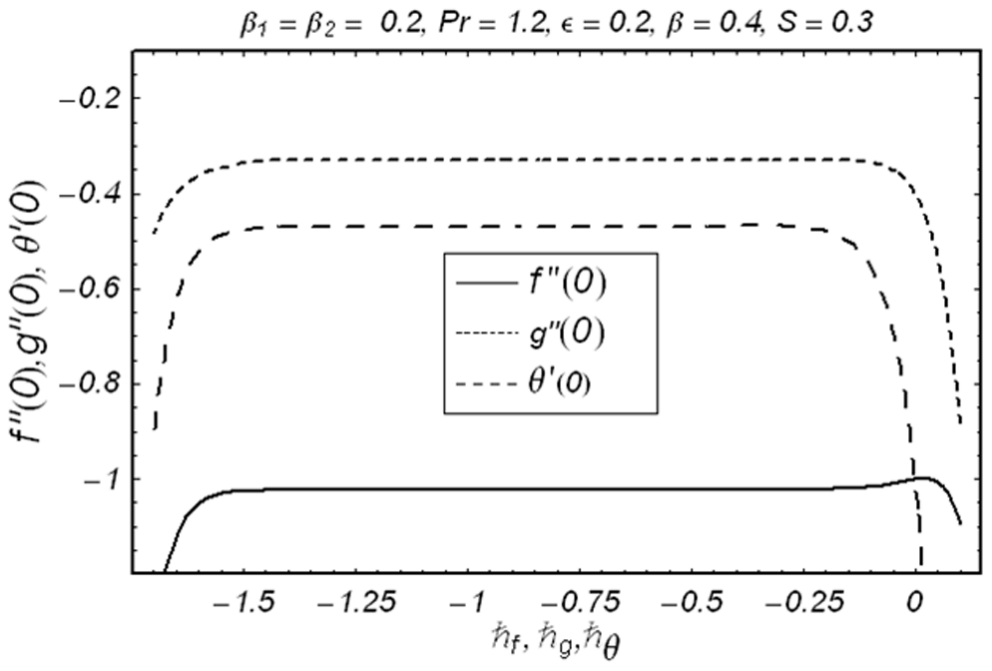
*h*-curves for the functions f(η), g(η) and θ(η).

Here the derived series (27)–(29) depend upon the auxiliary parameters 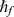, 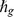 and 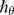 . These parameters are important to adjust and control the convergence of series solutions. The 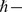 curves are sketched at 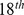 order of approximations just to determine the suitable ranges of 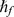, 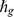 and 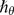 . Fig. 1 clearly showed that the range of admissible values of 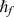, 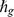 and 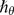 are 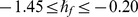, 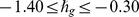 and 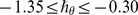. It is also observed that our series solutions converge in the whole region of 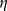 when 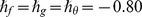 (see Table 1).

**Table 1:** Convergence of series solutions for different order of approximations when 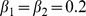, 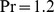, 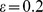, 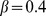, 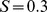 and 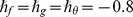.

Order of approximations	-f″(0)	-g″(0)	-θ′(0)
1	1.00480	0.32896	0.58000
5	1.02158	0.32915	0.46545
10	1.02157	0.32887	0.46899
17	1.02154	0.32887	0.46934
24	1.02154	0.32887	0.46931
30	1.02154	0.32887	0.46931
35	1.02154	0.32887	0.46931

**Figure 2:**
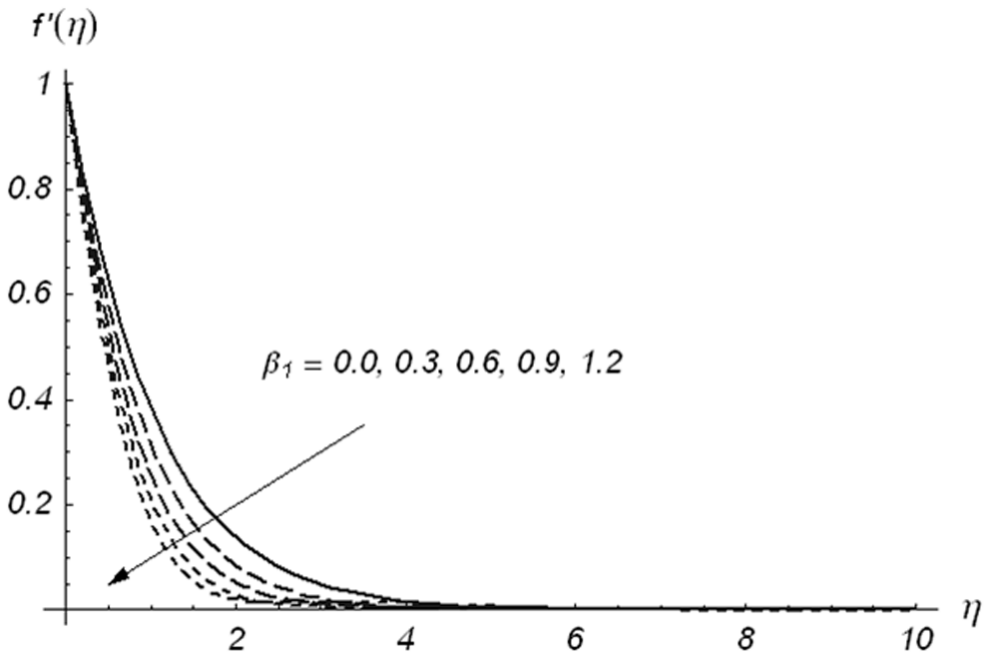
Variations of β_1_ on f′(η) when β_2_ = 0.3 and β = 0.5.

**Figure 3:**
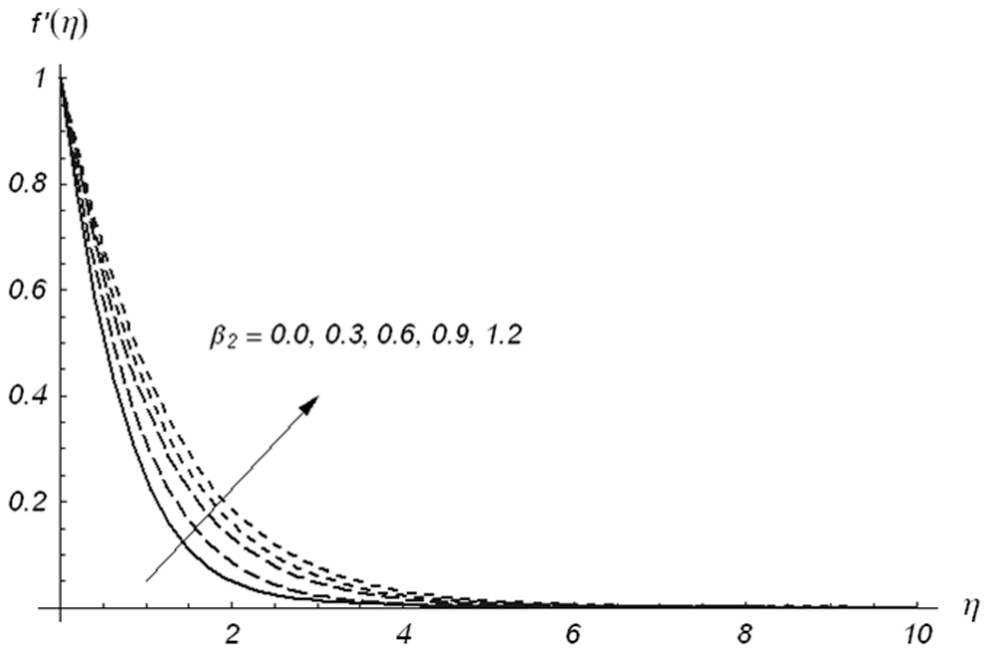
Variations of β_2_ on f′(η) when β_1_ = 0.3 and β = 0.5.

**Figure 4:**
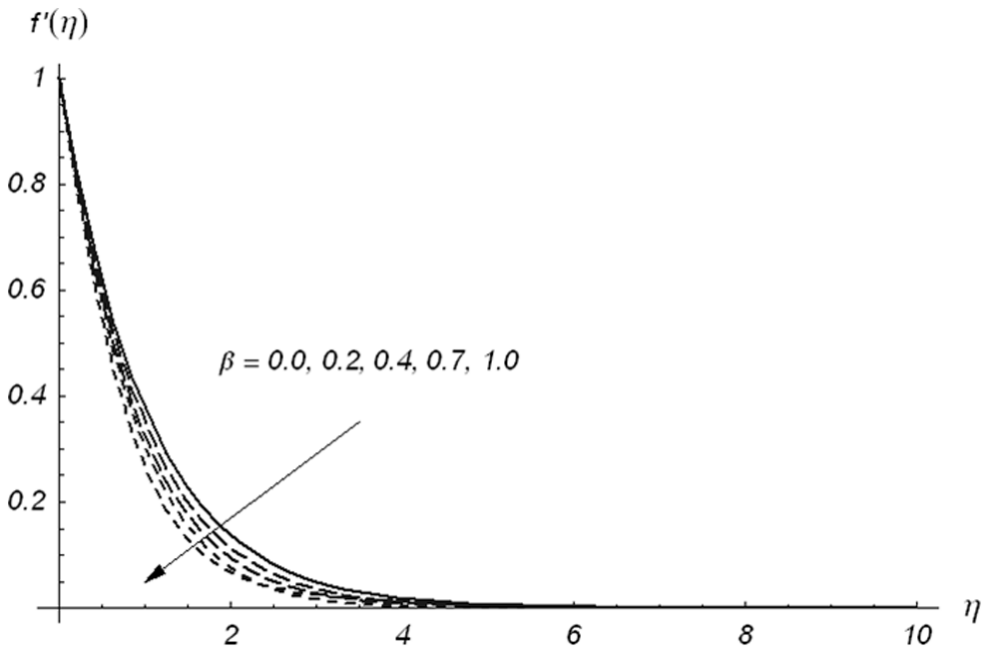
Variations of β on f′(η) when β_1_ = β_2_ = 0.3.

**Figure 5:**
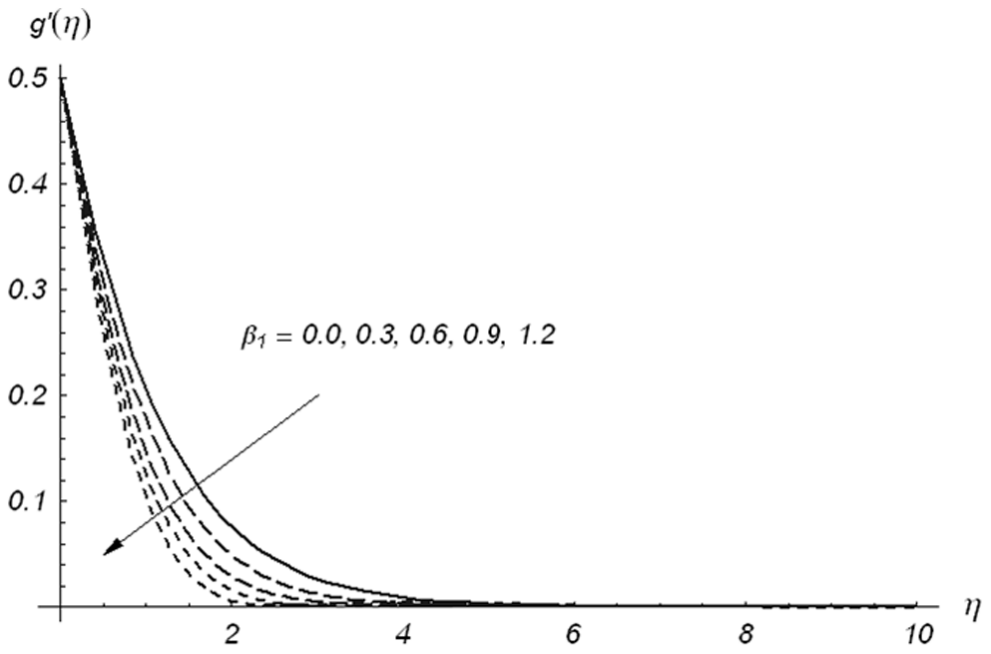
Variations of β_1_ on g′(η) when β_2_ = 0.3 and β = 0.5.

**Figure 6:**
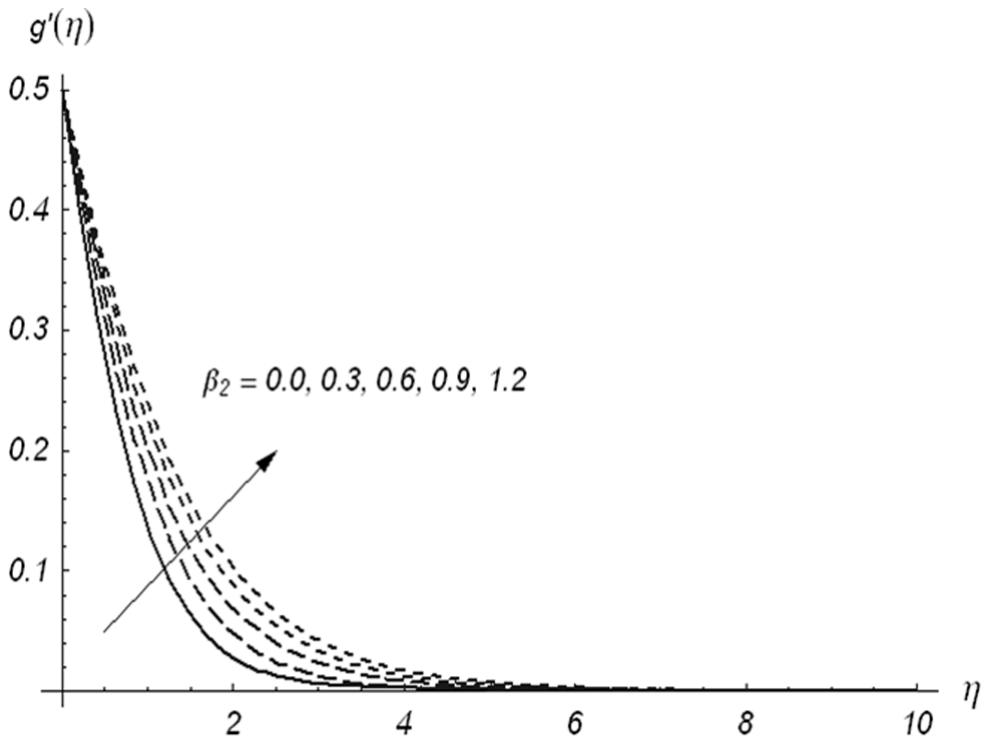
Variations of β_2_ on g′(η) when β_1_ = 0.3 and β = 0.5.

**Figure 7:**
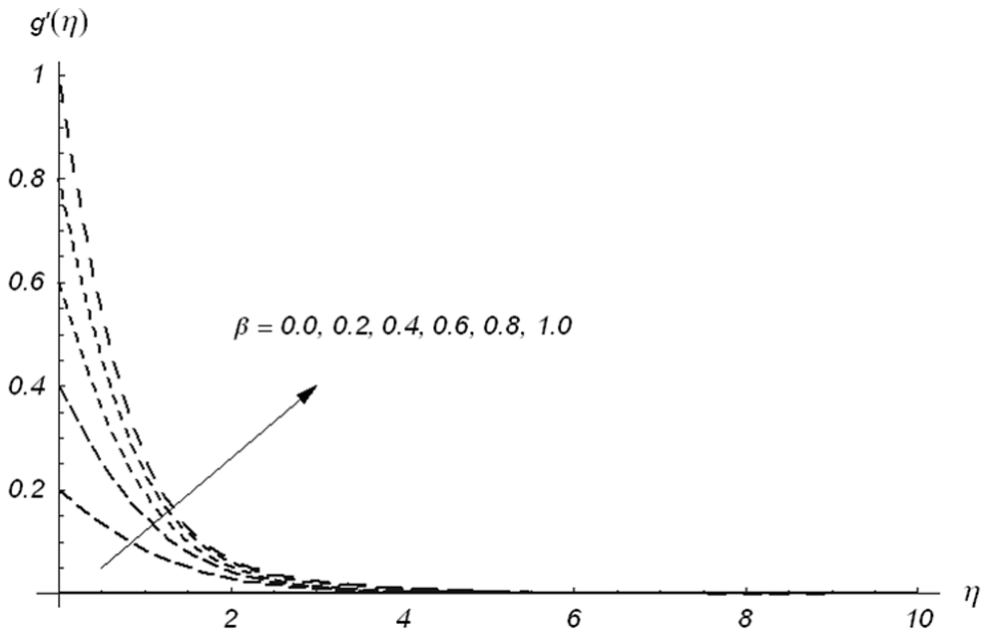
Variations of β on g′(η) when β_1_ = β_2_ = 0.3.

**Figure 8:**
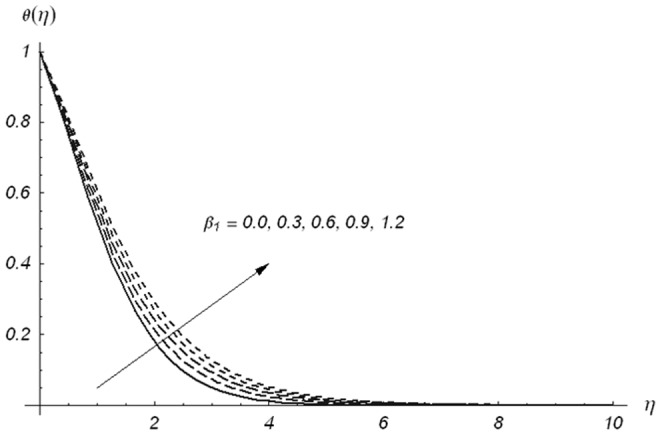
Variations of β_1_ on θ(η) when β_2_ = 0.3, β = 0.5, Pr = 1.2, S = 0.3 and ε = 0.2.

**Figure 9:**
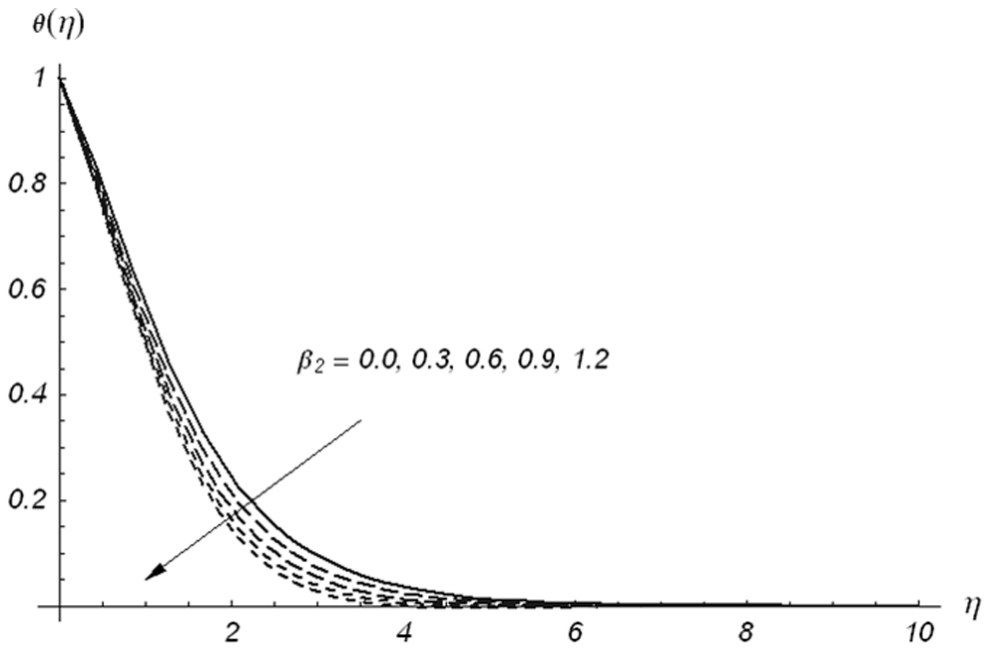
Variations of β_2_ on θ(η) when β_1_ = 0.3, β = 0.5, Pr = 1.2, S = 0.3 and ε = 0.2.

**Figure 10:**
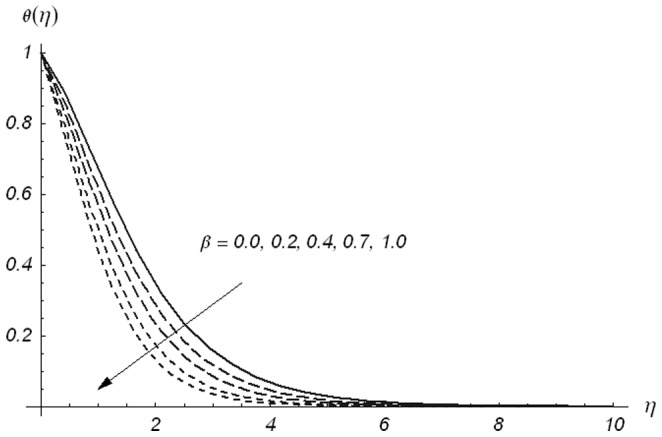
Variations of β on θ(η) when β_1_ = β_2_ = 0.5, Pr = 1.2, S = 0.3 and ε = 0.2.

**Figure 11:**
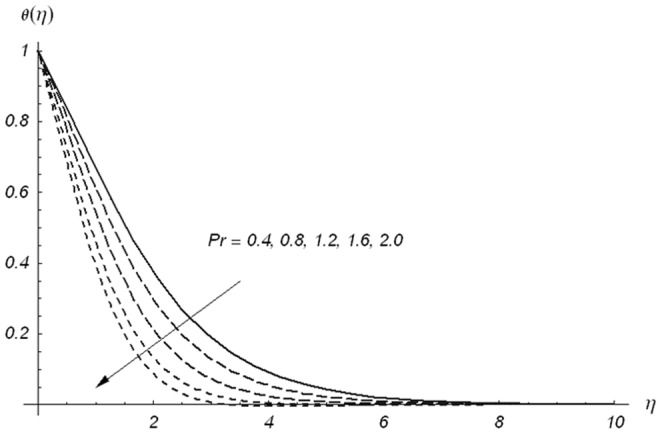
Variations of Pr on θ(η) when β_1_ = β_2_ = 0.5, β = 0.5, S = 0.3 and ε = 0.2.

**Figure 12:**
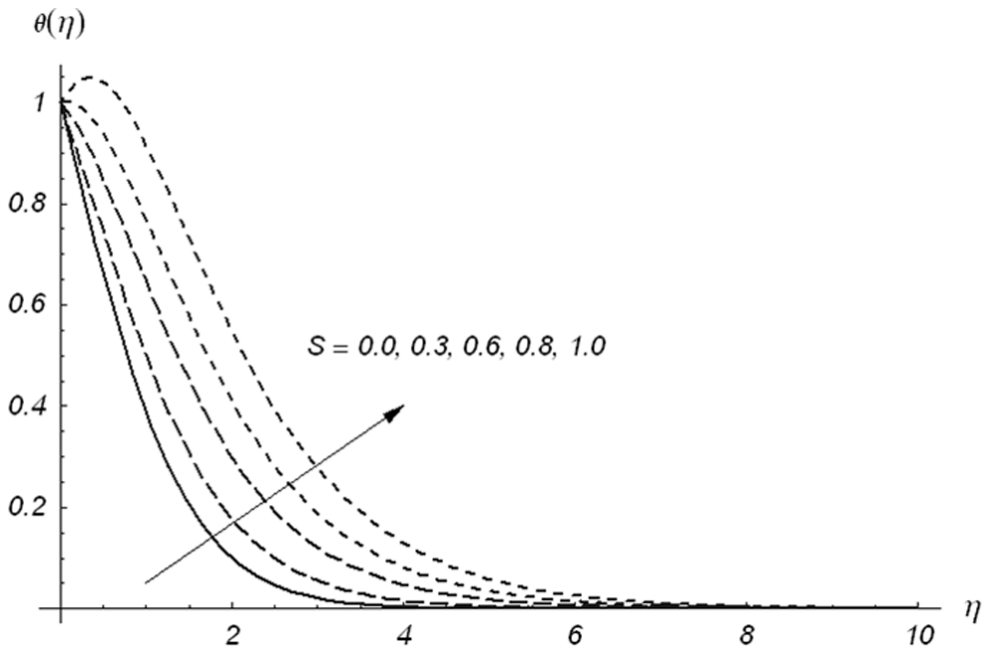
Variations of S on θ(η) when β_1_ = β_2_ = 0.5, β = 0.5, Pr = 1.2 and ε = 0.2.

**Figure 13:**
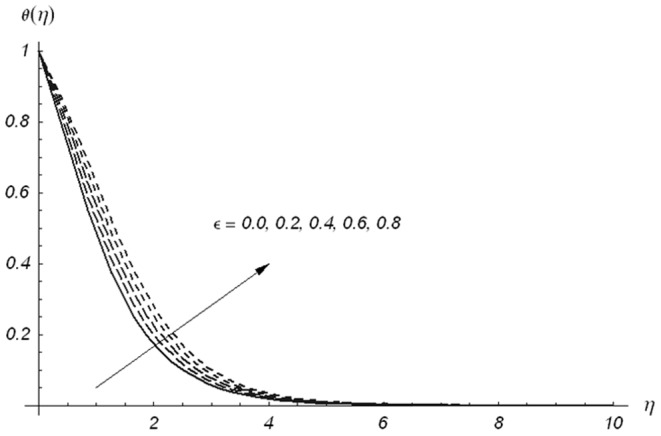
Variations of ε on θ(η) when β_1_ = β_2_ = 0.5, β = 0.5, Pr = 1.2 and S = 0.3.

The effects of Deborah numbers 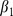, 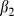 and ratio parameter 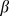 on the velocity component 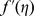 are displayed in the Figs. 2–4. Figs. 2 and 3 illustrate the variations of Deborah numbers on the velocity component 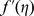. These Figs. clearly show that both 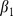 and 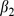 have reverse behaviors on the velocity component 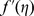. Physically, 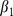 and 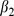 are dependent on the relaxation and retardation times, respectively. Increasing 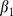 and 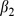 indicate that both relaxation and retardation times increase. It is well known fact that an increase in relaxation time decreases the velocity but velocity increases for larger retardation time. Due to this reason the dimensionless velocity component 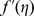 is decreased with an increase in 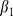 but a rise in the fluid velocity component 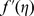 is seen when 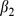 increases. The fluid velocity component 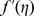 and momentum boundary layer thickness are reduced with the increasing values of ratio parameter 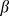 (see Fig. 4)  Figs. 5–7 describe the effects of 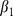, 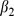 and 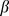 on the velocity component 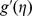. Fig. 5 depicts that the velocity component 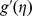 and its associated momentum boundary layer thickness are decreased with an increase in 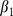. It can be noted from Fig. 6 that increasing values of 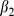 enhances the fluid velocity and momentum boundary layer thickness. Effects of 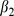 on the velocity components 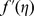 and 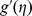 are similar in a qualitative sense (see Figs. 3 and 6). The velocity component 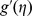 and momentum boundary layer thickness are increasing functions of 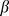. It is also observed from Fig. 7 that for 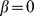, the variation in velocity component 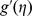 is zero and two-dimensional case for stretching surface is recovered. A comparison of Figs. 4 and 7 shows that the ratio parameter has quite opposite effects on the velocity components 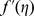 and 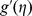. Actually, when 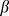 increases from zero, the lateral surface starts to move in the y-direction. Due to this argument, the velocity component 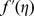 reduces while the velocity component 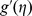 is increases. To examine the influence of 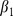, 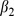, 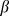, 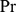 , 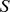 and 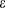 on the temperature 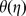, we have drawn Figs. 8–13. Fig. 8 depicts that the temperature increases for larger values of 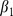. We concluded that the effect of 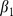 on the velocity components 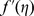, 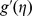 and temperature 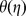 is reversed. The temperature and thermal boundary layer thickness become smaller for larger values of 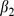. Fig. 9 leads to the conclusion that the temperature and thermal boundary layer thickness are decreasing functions of 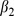. Fig. 10 shows that an increase in 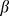 causes a reduction in temperature and thermal boundary layer thickness. The temperature and thermal boundary layer thickness are reduced for the increasing values of ratio parameter. From Fig. 11, we have seen that temperature field and thermal boundary layer thickness are smaller for larger values of Prandtl number. In fact larger Prandtl number corresponds to smaller thermal diffusivity and smaller thermal diffusivity provides a decrease in temperature and thermal boundary layer thickness. Fluids with smaller Prandtl number have higher thermal conductivities and thus have thicker thermal boundary layer structure. The main role of the Prandtl number is to adjust and control the rate of cooling fluids. Figs. 12 and 13 show the behaviors of 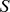 and 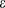 on the temperature field 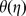. Increase in both 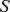 and 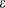 enhances the temperature and thermal boundary layer thickness. The difference we noted is that the temperature varies slowly and decays rapidly for 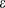 in comparison to 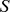. For S>0, the heat generation phenomenon occurs. This heat generation gives more heat to the fluid that corresponds to an increase in the temperature and thermal boundary layer thickness (see Fig. 12).

Table 1 provides the convergence values of series solutions. This Table clearly shows that 17th-order of approximations gives the convergent solutions for the velocities and 24th order deformations are required for the temperature. Table 2 shows the comparison for different values of 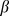 with homotopy perturbation method (HPM) and exact solutions. From this Table one can see that our series solutions have complete agreement with the previous HPM and exact solutions upto four decimal places. It is also examined that both -f″(0) and -g″(0) enhance for the increasing values of ratio parameter 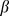. Numerical values of local Nusselt number -θ′(0) for different values of 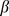, 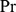, 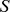 and 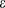 in both viscous and Oldroyd-B fluid cases are obtained in Table 3. We observed that the values of local Nusselt number for an Oldroyd-B fluid case are larger in comparison to the viscous fluid. It is also found that an increase in the values of 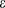 causes a reduction in the Nusselt number (see Table 3).

**Table 2:** Comparison for the different values of 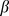 by HAM, HPM and exact solutions [30].

β	HPM [30]		Exact [30]		HAM	
	-f″(0)	-g″(0)	-f″(0)	-g″(0)	-f″(0)	-g″(0)
0.0	1.0	0.0	1.0	0.0	1.0	0.0
0.1	1.02025	0.06684	1.020259	0.66847	1.02026	0.06685
0.2	1.03949	0.14873	1.039495	0.148736	1.03949	0.14874
0.3	1.05795	0.24335	1.057954	0.243359	1.05795	0.24336
0.4	1.07578	0.34920	1.075788	0.349208	1.07578	0.34921
0.5	1.09309	0.46520	1.093095	0.465204	1.09309	0.46521
0.6	1.10994	0.59052	1.109946	0.590528	1.10994	0.59053
0.7	1.12639	0.72453	1.126397	0.724531	1.12639	0.72453
0.8	1.14248	0.86668	1.142488	0.866682	1.14249	0.86668
0.9	1.15825	1.01653	1.158253	1.016538	1.15826	1.01654
1.0	1.17372	1.17372	1.173720	1.173720	1.17372	1.17372

**Table 3:** Values of local Nusselt number 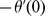 for the different values of the parameters 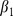, 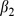, 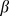, 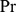, 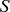 and 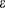 .

β	Pr	S	ε	-θ′(0)	
				β_1_ = β_2_ = 0.0	β_1_ = β_2_ = 0.3
0.0	1.3	0.3	0.2	0.15942	0.18436
0.6				0.54435	0.55510
1.0				0.67522	0.68113
0.5	0.8	0.3	0.2	0.22983	0.29794
	1.5			0.53178	0.58749
	2.0			0.68626	0.73129
0.5	1.3	0.0	0.2	0.76265	0.76872
		0.2		0.60416	0.61328
		0.5		0.20774	0.21046
0.5	1.3	0.3	0.0	0.59132	0.60313
			0.3	0.47028	0.48050
			0.6	0.38842	0.39777

## Conclusions

The three-dimensional flow of an Oldroyd-B fluid over a stretching surface is examined. Analysis with variable thermal conductivity and heat generation/absorption is conducted. The following conclusions can be drawn from the presented analysis.

- Deborah numbers 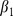 and 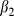 have quite opposite effects on the velocity component 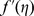.
- Effects of 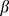 on the velocity components 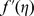 and 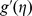 are opposite.
- Thermal boundary layer thickness and temperature of fluid are enhanced when there is an increase in 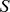.
- Numerical values of local Nusselt number are larger for an Oldroyd-B fluid than the viscous fluid.
- An increase in 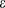 corresponds to a reduction in the values of Nusselt number.
- Results for three-dimensional flow of Maxwell fluid with variable thermal conductivity (which are not available yet) can be recovered by choosing 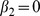.

The considered stretched flow of an Oldroyd-B fluid is important because it can be used in production of plastic sheet and extrusion of molten polymer through a slit die in polymer industry. This thermofluid problem involves significant heat transfer between the sheet and surrounding fluid. The extrudate in this mechanism starts to solidify as soon as it exits from the die and then sheet is collected by a wind-up roll upon solidification. Physical properties of the cooling medium, e.g., its thermal conductivity has pivotal role in such process. The success of whole operation closely depends upon the viscoelastic character of fluid above the sheet. The (drag) force required to pull the sheet can be determined by fluid viscosity. The variable thermal conductivity is quite common in polymeric and plastic industries. Electronics engineers rapidly are embracing thermally conductive plastics because they can absorb heat as well as most metals and can be modelled into intricate shapes and act as structural components as well. Especially the new generation of plastics is significant in components where heat build-up can degrade a conventional plastic. No one area gets overheated by spreading the heat load throughout the component. High thermally conductive polymers are useful in processes with dissipation of thermal energy. The knowledge of good thermal conductivity in modern thermal management composites is helpful in retaining typical properties of plastics such as low weight and electrical insulation. High energy generation rates within turbines or electronics require high thermal conductivity materials like copper and aluminium. The low thermal conductance materials such as polystyrene and alumina are useful in building construction or in furnaces for insulation purposes. It is hope that the present work will serve as a stimulus for needed experimental work on this problem.
