# Correction: Three-Dimensional Flow of an Oldroyd-B Fluid with Variable Thermal Conductivity and Heat Generation/Absorption

**DOI:** 10.1371/annotation/96dcc12e-4cef-4dcb-abf3-dac7dc0c4ac7

**Published:** 2014-01-06

**Authors:** Sabir Ali Shehzad, Ahmed Alsaedi, Tasawar Hayat, M. Shahab Alhuthali

In the Funding Statement, the number of the grant from the Deanship of Scientific Research of King Abdulaziz University is incorrect. The correct grant number is: 10-130/1434HiCi. 

